# Blood urea nitrogen to left ventricular ejection fraction ratio is a predictor of in-hospital outcomes in ST-elevation myocardial infarction patients: a case control study

**DOI:** 10.1186/s12872-025-05180-y

**Published:** 2025-09-29

**Authors:** Linfeng Xie, Jing Chen, Yuanzhu Li, Jian Shen, Xiang Li, Yuan Yang, Gang Liu, Yintao Chen, Bi Huang, Suxin Luo

**Affiliations:** Department of Cardiology, The First Affiliated Hospital of Chongqing Medical University, NO.1 Youyi Road, Yuzhong District, Chongqing, 400016 China

**Keywords:** Blood urea nitrogen, Left ventricular ejection fraction, ST-segment elevation myocardial infarction, In-hospital prognosis

## Abstract

**Background:**

The in-hospital mortality of ST-elevation myocardial infarction (STEMI) remains as high as 4–12%. Heart and kidney are closely linked, and both renal and cardiac function have been confirmed to be associated with the prognosis in patients with STEMI. This study intends to evaluate the prognostic value of blood urea nitrogen (BUN) to left ventricular ejection fraction (LVEF) ratio (BLR) in STEMI patients.

**Methods:**

From January 2015 to January 2023, 2435 consecutive STEMI patients were enrolled. The primary endpoint was in-hospital all-cause mortality and the second endpoint was major adverse cardiovascular events (MACE) including cardiovascular death, nonfatal stroke, and nonfatal myocardial infarction. The predictive value of BLR was compared with BUN, LVEF, traditional markers and scores (GRACE score and TIMI score) by receiver operating characteristic (ROC) curves, the area under the curve (AUC) were compared by DeLong test. Then patients were divided into two groups based on the cut-off value of BLR determined by Youden index and compared the in-hospital mortality and MACE. The association between BLR and endpoints was investigated by Cox regression.

**Results:**

Totally 2435 patients were included in our study, among which 90 (3.70%) patients died and 110 (4.52%) MACEs were collected. The non-survivors had significantly higher BUN level and lower LVEF value. The AUCs and DeLong test showed that the predictive value of BLR was significantly higher than BUN, LVEF, creatinine, N-terminal pro-brain natriuretic peptide (NT-proBNP), and troponin I but was comparable to GRACE score and TIMI scores. The optimal cut-off value of BLR was 12.54 with a sensitivity of 75.6% and a specificity of 67.6%. The in-hospital mortality and MACE was significantly higher in high BLR group (8.23% vs. 1.37% for in-hospital mortality and 9.44% vs. 1.99% for in-hospital MACE, all *p* < 0.001). After multivariable adjustment, BLR ≥ 12.54 was still independently associated with higher in-hospital mortality (HR = 2.464, 95%CI 1.448, 4.192, *p* < 0.001) and MACE (HR = 1.850, 95%CI 1.175, 2.911, *p* = 0.008).

**Conclusion:**

BLR is an important prognostic index to identify patients at high risk of in-hospital prognosis in STEMI patients and the prognostic value was comparable to or even higher than traditional prognostic scores.

**Trial registration:**

ChiCTR1900028516 (http//www.chictr.org.cn).

## Background

Worldwide, ischaemic heart disease is one of the most common causes of death and its incidence is still increasing [1]. Although the widespread use of reperfusion therapy, primary percutaneous coronary intervention (PCI), and modern antithrombotic therapy have significantly improved the long-term outcome in patients with acute ST-elevation myocardial infarction (STEMI) [2–4], the in-hospital mortality of unselected patients with STEMI was still high, between 4–12% [5]. Therefore, after reperfusion treatment, it is important to identify patients at high risk of subsequent events such as reinfarction or death. Currently, several risk scores have been developed based on readily identifiable parameters in the acute phase of acute myocardial infarction (AMI) [6], among which the TIMI score [7] and GRACE score [8] are widely used in clinical practice. However, their clinical use needs complex algorithms and the inclusion of multiple metrics.

Previous studies have shown both renal function and cardiac function were associated with the outcome of STEMI patients [1]. Pathophysiologically, heart and kidneys are two closely related organs and interact with each other, so called cardiorenal syndrome (CRS) [9]. Therefore, combining renal and cardiac function together could provide more accurate prognostic value for patients with AMI. Blood urea nitrogen (BUN) is a surrogate of renal dysfunction and was shown to be superior to creatinine for the evaluation of prognosis in AMI patients [10]; left ventricular ejection fraction (LVEF) is the most commonly used index to reflect the cardiac function [11]. In recent years, a new index, BUN to LVEF ratio (BLR), has been proposed to evaluate the outcome in patients with cardiovascular diseases [12–15]. However, no studies, to the best of our knowledge, have been explicitly designed to assess the short term prognostic value of BLR in STEMI patients. Accordingly, the present study aimed to test the utility of BLR as a simple but effective tool for risk stratification for STEMI patients.

## Methods

### Study design

This study was a retrospective, single-center study that involved 2435 patients who were diagnosed with STEMI from January 2015 and January 2023 with the aim to evaluate the prognostic value of BLR. The study protocol was approved by the Ethics Committee of the First Affiliated Hospital of Chongqing Medical University (number: 2019 − 148, date: 2019-07-15) and complied with the Declaration of Helsinki.

### Inclusion and exclusion criteria

Inclusion criteria: aged 18 years or older and diagnosed with STEMI at the time of admission from January 2015 and February 2023. STEMI was defined as follows: chest pain or equivalent symptoms in combination with dynamic electrocardiographic changes consistent with STEMI, and increased serum troponin I (TnI).

Exclusion criteria: BUN or LVEF value were unavaiable; those who did not receive coronary angiography.

### Treatment and BLR measurement

After admission, all patients received an overall evaluation. If there was no contraindication and the patients agreed, emergent coronary angiography and PCI were recommended. After the intervention procedure, patients were sent to the coronary care unit for electrocardiogram monitoring and further management was administered according to the guidelines [16, 17].

The echocardiogram was performed within 24 h after admission. Left ventricular volumes were measured by Simpson’s disk method and LVEF was calculated according to the American Society of Echocardiography protocol [18]. BUN were measured at admission, the reference range of our laboratory for BUN was 3.6–9.5 mmol/L (Ortho-Clinical Diagnostics, Inc, US). To ensure reliability and accuracy of the data, baseline characteristics, auxiliary examinations, and treatment were collected by experienced clinicians from the computerized patient record system.

### Study endpoint

The primary study endpoint of this study was in-hospital all-cause mortality and the second study endpoint was major adverse cardiovascular events (MACE) including cardiovascular mortality, nonfatal stroke, and nonfatal myocardial infarction (MI).

### Calculation of TIMI score and GRACE score

The calculation of TIMI score includes the following variables [7]: age, historical diabetes mellitus/hypertension or angina, systolic blood pressure, heart rate, Killip class, weight, anterior ST segment deviation or left bundle branch block, and time to treatment. The variables in GRACE score [8] includes age, Killip class, systolic blood pressure, ST segment deviation, cardiac arrest at admission, serum creatinine, raised cardiac markers, and heart rate.

### Statistical analysis

Continuous variables with normal distribution were presented in mean value and standard deviations, otherwise as the median value and inter-quartile range (25th and 75th), and two independent sample t-test or Mann-Whitney U test were used for the comparisons between groups respectively. Categorical variables were presented in numbers and percentages, and Chi-Square test test or Fisher test was employed. The prognostic value of BUN, LVEF, creatinine, N-terminal pro-brain natriuretic peptide (NT-proBNP), TnI, GRACE score, TIMI score and BLR was evaluated by the receiver operating characteristic curve (ROC) and the area under the curve (AUC) for in-hospital mortality was calculated. The AUCs of those indexes were further compared by DeLong test. The optimal cut-off value of BLR was determined by Youden index, then patients were divided into two groups based on the cut-off value. Univariate and multivariate Cox regression models were constructed to confirm whether there was an independent relationship between BLR and clinical outcomes, based on the previous studies and taking the clinical relevance and model stability into consideration. The factors in the model included age, sex, admission systolic blood pressure, admission heart rate, NT-proBNP, TnI, white blood cell, Killip class > I, TIMI score, GRACE score, the use of beta-blockers and angiotensin-converting enzyme inhibitors or angiotensin receptor blockers (ACEI/ARB), in which NT-proBNP and TnI were changed into categorical variables by the cut-off value of them determined by Youden index. For a more detailed analysis of the association between BLR and in-hospital all-cause mortality and MACE in patients with different clinical profiling, subgroups were analyzed based on age (≥ vs. <75 years old), gender, hypertension, diabetes, renal dysfunction, BUN (≥ vs. <5.8mmol/L), LVEF (≥ vs. <50%), Killip class (> vs. =1), TIMI score (low or medium vs. high), and anterior MI. A two-tailed p-value of < 0.05 was considered statistically significant and All statistical analyses were carried out using the SPSS statistical software, version 25.0 (IBM, USA), MedCalc statistical software 19.2.6, and GraphPad Prism 8.4.3.

## Results

From January 2015 to January 2023, a total of 2661 consecutive STEMI patients admitted, among which 226 patients had incomplete data or did not receive coronary angiography, the remaining 2435 patients were included in this study. The mean age of this cohort was 63 (SD, 13) years and 1947 (80.0%) were male. During hospitalization, a total of 90 (3.70%) patients died and 110 (4.52%) MACEs occurred.

**Fig. 1:**
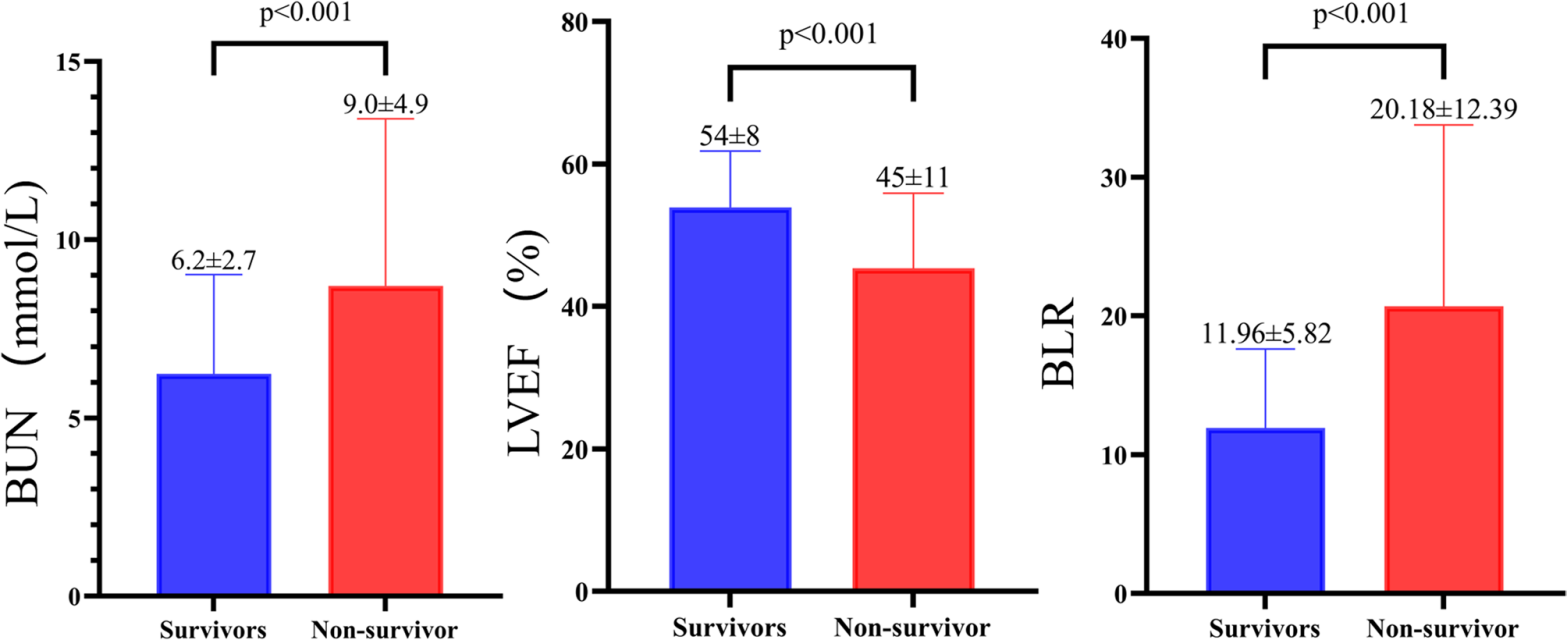
The comparision of BUN, LVEF, and BLR between survivors and non-survivor. (BUN: Blood urea nitrogen; LVEF: Left ventricular ejection fraction; BLR: Blood urea nitrogen to left ventricular ejection fraction ratio)

Compared with survivors, non-survivors had a higher level of BUN (9.0 ± 4.9 vs. 6.2 ± 2.7 mmol/L, *p* < 0.001, Fig. 1), lower LVEF (45 ± 11% vs. 54 ± 8%, *p* < 0.001, Fig. 1) and higher BLR (11.96 ± 5.82 vs. 20.18 ± 12.39, *p* < 0.001, Fig. 1).

**Fig. 2:**
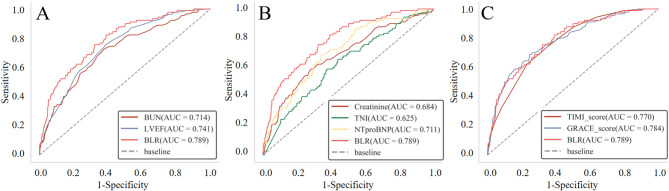
The ROC of prognostic indexes for in-hospital mortality. (**A**) The ROC of BUN, LVEF and BLR for predicting in-hospital mortality. (**B**) The ROC of TNI, NT-proBNP, creatinine and BLR for predicting in-hospital mortality. (**C**) The ROC of GRACE score, TIMI score and BLR for predicting in-hospital mortality. (BUN: Blood urea nitrogen; LVEF: Left ventricular ejection fraction; BLR: Blood urea nitrogen to left ventricular ejection fraction ratio; TNI: Troponin I; NT-proBNP: N-Terminal Pro-Brain Natriuretic Peptide)

**Fig. 3:**
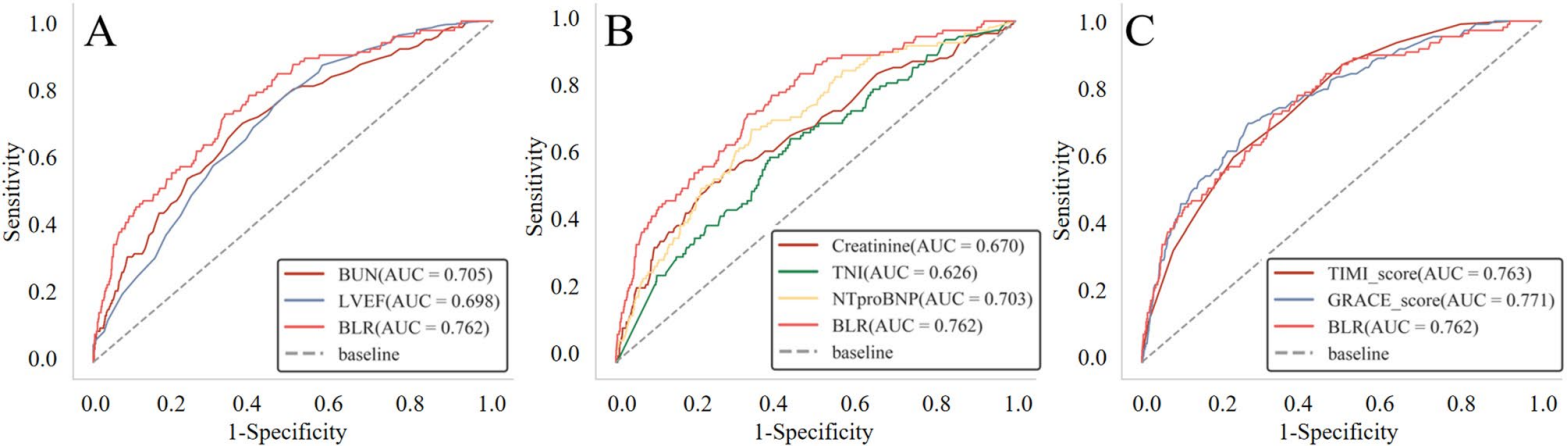
The ROC of prognostic indexes for MACE. (**A**) The ROC of BUN, LVEF and BLR for predicting in-hospital mortality. (**B**) The ROC of TNI, NT-proBNP, creatinine and BLR for predicting in-hospital mortality. (**C**) The ROC of GRACE score, TIMI score and BLR for predicting in-hospital mortality. (MACE: major adverse cardiovascular events, BUN: Blood urea nitrogen; LVEF: Left ventricular ejection fraction; BLR: Blood urea nitrogen to left ventricular ejection fraction ratio; TNI: Troponin I; NT-proBNP: N-Terminal Pro-Brain Natriuretic Peptide)

The ROC of BUN, LVEF, creatinine, NT-proBNP, TnI, GRACE score, TIMI score, and BLR for predicting in-hospital mortality and MACE were presented in Figs. 2 and 3. The AUC of BUN, LVEF, creatinine, NT-proBNP, TnI, GRACE score, TIMI score and BLR for in-hospital mortality were 0.714, 0.741, 0.684, 0.711, 0.625, 0.784, 0.770, and 0.789, respectively; for MACE AUCs were 0.705, 0.698, 0.670, 0.703, 0.626, 0.771, 0.763, and 0.762, respectively, indicating BLR had highest prognostic power for in-hospital mortality and MACE. DeLong test showed that the predictive value of BLR was significantly higher than BUN, LVEF, creatinine, NT-proBNP, and TnI (all *p* < 0.05), but was comparable to GRACE score and TIMI scores (all *p* > 0.05). The optimal cut-off value of BLR for predicting in-hospital All-cause mortality was 12.54 determined by Yonden index with a sensitivity of 75.6% and a specificity of 67.6%. Then patients were divided into two groups, BLR < 12.54 and BLR ≥ 12.54 for further analysis.

Table 1 showed the baseline characteristics and treatment of the 2 groups divided according to BLR cut-off value. Patients with higher BLR tended to be older (67 ± 12 vs. 61 ± 12 yeas, *p* < 0.001), had lower body mass index (23.74 ± 3.43 vs. 24.54 ± 3.43 kg/m^2^, *p* < 0.001), and had a higher proportion of previous MI (6.1% vs. 4.0%, *p* = 0.027), PCI (5.4% vs. 3.1%, *p* = 0.005), hypertension (59.2% vs. 49.3%, *p* < 0.001), diabetes (33.5% vs. 23.3%, *p* < 0.001), renal dysfunction (32.6% vs. 6.2%, *p* < 0.001), and previous stroke (6.8% vs. 4.4%, *p* = 0.010). At admission, more patients in higher BLR group presented with higher Killip class, lower blood pressure, higher heart rate, and cardiac shock (all *p* < 0.05).

**Table 1:** Comparison of baseline characteristics divided by BLR

	Total (*n* = 2435)	BLR ≥ 12.54 (*n* = 826)	BLR < 12.54 (*n* = 1609)	*P* value
Demographics				
Age (years)	63 ± 13	67 ± 12	61 ± 12	< 0.001
Male (*n*, %)	1947 (80.0%)	650 (78.7%)	1297 (80.6%)	0.263
BMI (Kg/m^2^)	24.27 ± 3.45	23.74 ± 3.43	24.54 ± 3.43	< 0.001
Co-morbidities (*n*, %)				
Previous MI	115 (4.7%)	50 (6.1%)	65 (4.0%)	0.027
Previous PCI	95 (3.9%)	45 (5.4%)	50 (3.1%)	0.005
Hypertension	1282 (52.6%)	489 (59.2%)	793 (49.3%)	< 0.001
Diabetes mellitus	652 (26.8%)	277 (33.5%)	375 (23.3%)	< 0.001
Dislipidemia	370 (15.2%)	116 (14.0%)	254 (15.8%)	0.257
Renal dysfunction^a^	368 (15.1%)	269 (32.6%)	99 (6.2%)	< 0.001
Previous stroke	126 (5.2%)	56 (6.8%)	70 (4.4%)	0.010
Current smoker	1613 (66.2%)	529 (64.0%)	1084 (67.4%)	0.100
Clinical presentation (*n*, %)				
Cardiac arrest before admission	108 (4.4%)	38 (4.6%)	70 (4.4%)	0.777
Killip class				< 0.001
I	1841 (75.6%)	534 (64.6%)	1307 (81.2%)	
II	278 (11.4%)	129 (15.6%)	149 (9.3%)	
III	55 (2.3%)	34 (4.1%)	21 (1.3%)	
IV	261 (10.7%)	129 (15.6%)	132 (8.2%)	
Cardiogenic shock	196 (8.0%)	144 (13.8%)	82 (5.1%)	< 0.001
Admission vital signs				
Systolic blood pressure (mmHg)	125 ± 25	122 ± 26	127 ± 25	< 0.001
Diastolic blood pressure (mmHg)	78 ± 17	76 ± 17	79 ± 16	< 0.001
Heart rate (bpm)	82 ± 18	83 ± 20	82 ± 17	0.031
Location of MI (*n*, %)				
Anterior MI	1281 (52.6%)	428 (51.3%)	853 (53.0%)	0.575
Lateral MI	267 (11.0%)	104 (12.6%)	161 (10.1%)	0.066
Inferior MI	1200 (49.3%)	419 (50.7%)	781 (48.5%)	0.307
Right ventricle MI	262 (10.8%)	95 (11.5%)	167 (10.4%)	0.398
Posterior MI	324 (13.3%)	119 (14.4%)	205 (12.7%)	0.252
Culprit vessel (*n*, %)				
LM	12 (0.5%)	9 (1.1%)	3 (0.2%)	0.003
LAD	1265 (52.0%)	417 (50.5%)	848 (52.7%)	0.292
LCX	278 (11.4%)	93 (11.3%)	185 (11.5%)	0.857
RCA	940 (38.6%)	329 (39.8%)	611 (38.0%)	0.379
Laboratory findings				
Troponin I (ng/mL)	3.12 (0.30, 14.60)	4.24 (0.50, 18.10)	2.58 (0.25, 12.90)	< 0.001
NT-proBNP (pg/mL)	137 (39, 505)	270 (79, 1155)	98 (31, 320)	< 0.001
D-dimer (ng/mL)	285 (100, 659)	485 (189, 1020)	217 (100, 487)	< 0.001
White blood cell counts (× 10^9^/L)	11.31 ± 3.87	11.68 ± 4.24	11.11 ± 3.65	< 0.001
Hemoglobin (g/L)	138 ± 19	134 ± 20	141 ± 19	< 0.001
Blood urea nitrogen (mmol/L)	6.3 ± 2.9	8.7 ± 3.8	5.1 ± 1.1	< 0.001
Creatinine (µmol/L)	75 (63, 90)	87 (71, 115)	71 (60, 82)	< 0.001
LDL (mmol/L)	2.84 ± 0.93	2.72 ± 0.92	2.91 ± 0.93	< 0.001
HDL (mmol/L)	1.09 ± 0.31	1.12 ± 0.33	1.07 ± 0.29	< 0.001
Echocardiography findings				
LVEF (%)	54 ± 8	49 ± 9	56 ± 6	< 0.001
LVEDD (mm)	49 ± 5	50 ± 6	48 ± 4	< 0.001
Regional wall motion abnormality (*n*, %)	2092 (88.7%)	728 (91.4%)	1364 (87.4%)	0.005
Ventricular aneurysm (*n*, %)	105 (4.4%)	63 (7.9%)	42 (2.7%)	< 0.001
Medication use in hospital (*n*, %)				
Aspirin	2358 (96.8%)	794 (96.1%)	1564 (97.2%)	0.150
P2Y12 receptor inhibitors	2404 (98.7%)	812 (98.3%)	1592 (98.9%)	0.183
Statins	2398 (98.5%)	803 (97.2%)	1595 (99.1%)	< 0.001
Beta-blockers	1323 (54.3%)	380 (46.0%)	943 (58.6%)	< 0.001
ACEI/ARB	815 (33.5%)	218 (26.4%)	597 (37.1%)	< 0.001
Anticoagulant drug	101 (4.1%)	49 (5.9%)	52 (3.2%)	0.002
PPI	2159 (88.7%)	754 (91.3%)	1405 (87.3%)	0.004
Percutaneous coronary intervention	2292 (94.1%)	773 (93.6%)	1519 (94.4%)	0.414
TIMI score	4.84 ± 2.52	5.73 ± 2.51	4.38 ± 2.40	< 0.001
GEACE score	128 (106, 155)	145 (120, 171)	120 (101, 145)	< 0.001

*BLR* Blood urea nitrogen to left ventricular ejection fraction ratio, *BMI* Body mass index, *MI* Myocardial infarction, *PCI* Percutaneous coronary intervention, *LM* Left main artery, *LAD* Left anterior descending artery, *LCX* Left circumfex artery, *RCA* Right coronary artery, *NT-proBNP* N-terminal pro-brain natriuretic peptide, *LDL* Low-density lipoprotein, *HDL* High-density lipoprotein, *LVEF* Left ventricular ejection fraction, *LVEDD* Left ventricular end-diastolic dimension, *ACEI* Angiotensin-converting enzyme inhibitor, *ARB* Angiotensin-converting receptor blocker, *PPI* Proton pump inhibitor)

a: Renal dysfunction: estimate glomerular filtration rate< 60mL/(min*1.73^2)

The location of MI on electrocardiogram was similar between the two groups (all *p* > 0.05). On coronary angiography, the distribution of culprit vessels were similar except that left main coronary artery was relatively more common in patients with higher BLR (1.1% vs. 0.2%, *p* = 0.003). The comparison of laboratory findings revealed that patients with higher BLR had a higher level of TnI, NT-proBNP, D-dimer, white blood cell counts, BUN, creatinine, and high-density lipoprotein, but had a lower level of hemoglobin and low-density lipoprotein (all *p* < 0.05). The echocardiography showed that patients in higher BLR group had lower LVEF (49 ± 9% vs. 56 ± 6%, *p* < 0.001), larger left ventricular end-diastolic dimension (50 ± 6 vs. 48 ± 4 mm, *p* < 0.001), and more regional wall motion abnormality (91.4% vs. 87.4%, *p* = 0.005), ventricular aneurysm (7.9% vs. 2.7%, *p* < 0.001). During hospitalization, statins, beta-blockers, ACEI/ARB were more prescribed to patients with lower BLR, while proton pump inhibitor and anticoagulant drugs were more used in patients with higher BLR (all *p* < 0.05). The percentage of PCI was comparable between the two groups (*p* > 0.05). The mean TIMI score and GRACE score in patients with higher BLR was 5.73 and 145, respectively, and were significantly higher than in patients with lower BLR (all *p* < 0.001).

**Fig. 4:**
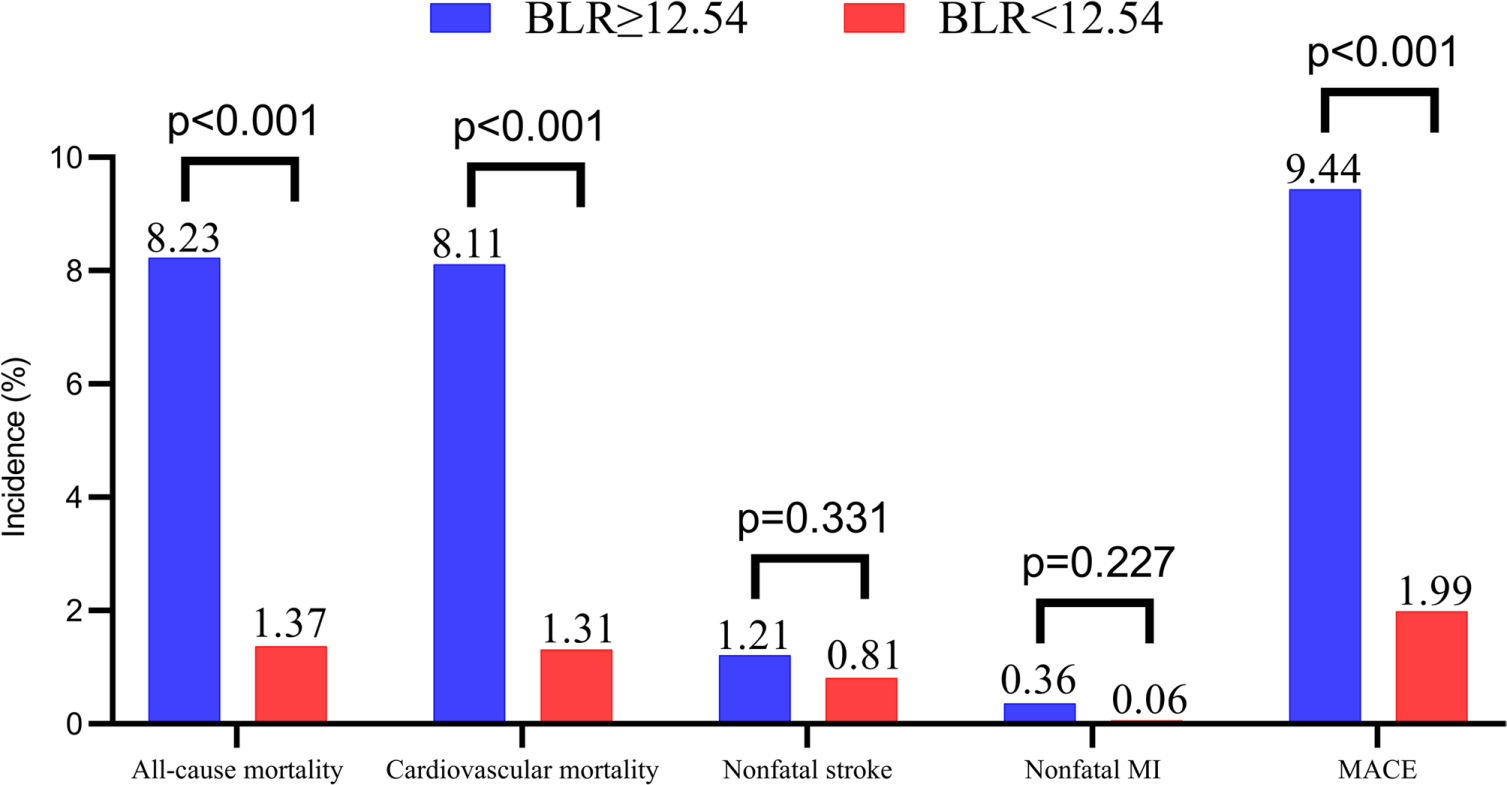
The in-hospital major adverse events of patients with BLR ≥ 12.54 and < 12.54. (BLR: Blood urea nitrogen to left ventricular ejection fraction ratio, MI: myocardial infarction, MACE: major adverse cardiovascular events)

**Fig. 5:**
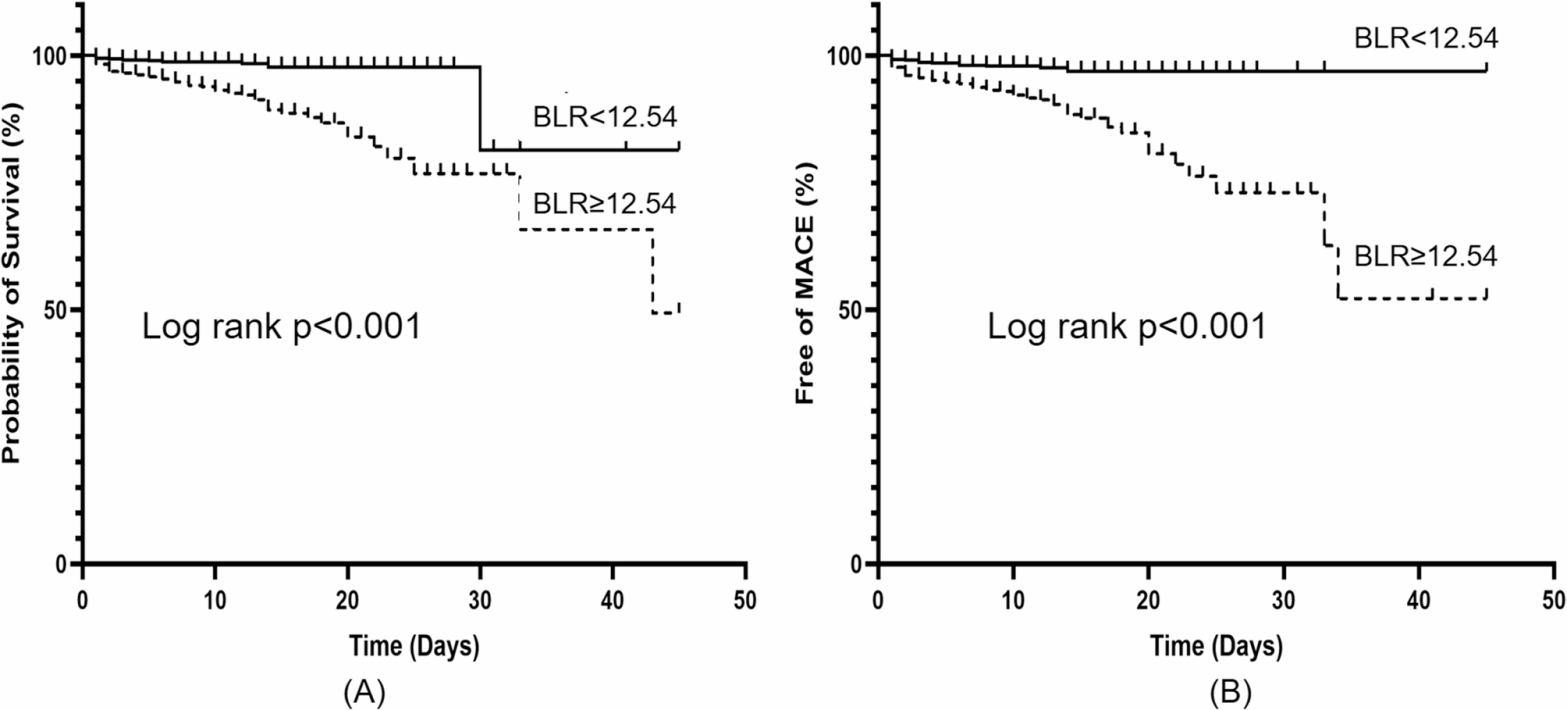
Kaplan-Meier curves for in-hospital all-cause mortality (**A**) and MACE (**B**) in STEMI patients (MACE: major adverse cardiovascular events, STEMI: ST-elevation myocardial infarction)

Figure 4 showed the in-hospital outcomes between patients with high and low BLR. The in-hospital mortality and MACE were significantly higher in BLR ≥ 12.54 group (8.23% vs. 1.37% for in-hospital mortality, 9.44% vs. 1.99% for MACE, all *p* < 0.001). The cardiovascular mortality was significantly higher in high BLR group (8.11% vs. 1.31%, *p* < 0.001), while the nonfatal stroke and nonfatal MI were similar between the two groups (1.21% vs. 0.81%, *p* = 0.331 for nonfatal stroke and 0.36% vs. 0.06%, *p* = 0.227 for nonfatal MI, respectively). Figure 5 displays the K-M curves of the two group patients and it revealed the cumulative survival and free of MACE in patients with higher BLR were significantly lower than that in patients with lower BLR (all Log rank *p* < 0.001).

Table 2 displayed the results from univirate and multivariate Cox regression for in-hospital mortality. Compared with BLR < 12.54, BLR ≥ 12.54 was associated with Almost 4-fold increased risk of in-hospital all-cause mortality (HR = 4.773, 95%CI 2.935, 7.762, *p* < 0.001). After multivariate adjustment, BLR ≥ 12.54 was still an independent prognostic factor for in-hospital mortality (HR = 2.464, 95%CI 1.448, 4.192, *p* < 0.001). Other independent prognostic factors included heart rate (HR = 1.018, 95%CI 1.007, 1.029, *p* < 0.001), white blood cell (HR = 1.071, 95%CI 1.023, 1.121, *p* = 0.003), NT-proBNP > 265.5 pg/mL (HR = 1.949, 95%CI 1.189, 3.195, *p* = 0.008), TNI > 4.50 ng/mL (HR = 1.774, 95%CI 1.128, 2.788, *p* = 0.013), and GRACE score (HR = 1.010, 95%CI 1.000, 1.020, *p* = 0.041).

**Table 2:** The univariate and multivariate Cox regression analysis of in-hospital mortality

Predictors for in-hospital mortality	Univariate analysis HR (95%CI) *P*		Multivariate analysis HR (95%CI) *P*	
Age	1.051 (1.031, 1.071)	< 0.001		
Male	1.830 (1.183, 2.831)	0.007		
Admission heart rate	1.026 (1.017, 1.035)	< 0.001	1.018 (1.007, 1.029)	< 0.001
Admission SBP	0.985 (0.977, 0.992)	< 0.001		
NT-proBNP > 265.5 (pg/mL)	3.361 (2.131, 5.303)	< 0.001	1.949 (1.189, 3.195)	0.008
Troponin I > 4.50 (ng/mL)	2.112 (1.371, 3.254)	0.001	1.774 (1.128, 2.788)	0.013
White blood cell (*10^9/L)	1.105 (1.065, 1.146)	< 0.001	1.071 (1.023, 1.121)	0.003
Killip Class > I	3.502 (2.286, 5.365)	< 0.001		
GRACE score	1.019 (1.015, 1.023)	< 0.001	1.010 (1.000, 1.020)	0.041
TIMI score	1.375 (1.271, 1.488)	< 0.001		
Beta-blockers	0.491 (0.319, 0.757)	0.001		
ACEI/ARB	0.412 (0.233, 0.730)	0.002		
BLR (≥ 12.54)	4.773 (2.935, 7.762)	< 0.001	2.464 (1.448, 4.192)	< 0.001

*SBP* Systolic blood pressure, *NT-proBNP* N-terminal-pro-B-type-natriuretic-peptide, *ACEI/ARB* Angiotensin-converting enzyme inhibitors or Angiotensin receptor blockers, *BLR* Blood urea nitrogen to left ventricular ejection fraction ratio

Table 3 showed the association of BLR with in-hospital MACE. Compared with BLR < 12.54, BLR ≥ 12.54 was associated with Almost 3-flold increased risk of in-hospital MACE (HR = 3.866, 95%CI 2.548, 5.865, *p* < 0.001). Similarly, BLR ≥ 12.54 was independently associated with increased risk of in-hospital MACE after multivariate adjustment (HR = 1.850, 95%CI 1.175, 2.911, *p* = 0.008). Other independent factors associated with in-hospital MACE included age (HR = 1.041, 95%CI 1.012, 1.070, *p* = 0.005), heart rate (HR = 1.021, 95%CI 1.010, 1.031, *p* < 0.001), white blood cell (HR = 1.063, 95%CI 1.020, 1.108, *p* = 0.004), NT-proBNP > 265.5 pg/mL (HR = 1.941, 95%CI 1.248, 3.018, *p* = 0.003), and TNI > 4.50 ng/mL (HR = 1.848, 95%CI 1.231, 2.775, *p* = 0.003).

**Table 3:** The univariate and multivariate Cox regression analysis of in-hospital MACE

Predictors for in-hospital MACE	Univariate analysis HR (95%CI) *P*		Multivariate analysis HR (95%CI) *P*	
Age	1.054 (1.036, 1.072)	< 0.001	1.041 (1.012, 1.070)	0.005
Male	1.590 (1.061, 2.384)	0.025		
Admission heart rate	1.026 (1.018, 1.035)	< 0.001	1.021 (1.010, 1.031)	< 0.001
Admission SBP	0.987 (0.980, 0.995)	0.001		
NT-proBNP > 265.5 (pg/mL)	3.335 (2.217, 5.017)	< 0.001	1.941 (1.248, 3.018)	0.003
Troponin I > 4.50 (ng/mL)	2.175 (1.471, 3.217)	< 0.001	1.848 (1.231, 2.775)	0.003
White blood cell (*10^9/L)	1.095 (1.056, 1.132)	< 0.001	1.063 (1.020, 1.108)	0.004
Killip Class > I	3.386 (2.307, 4.969)	< 0.001		
GRACE score	1.018 (1.014, 1.022)	< 0.001		
TIMI score	1.360 (1.267, 1.459)	< 0.001		
Beta-blockers	0.440 (0.296, 0.653)	< 0.001		
ACEI/ARB	0.458 (0.279, 0.752)	0.002		
BLR (≥ 12.54)	3.866 (2.548, 5.865)	< 0.001	1.850 (1.175, 2.911)	0.008

*MACE* Major adverse cardiovascular events, *SBP* Systolic blood pressure, *NT-proBNP* N-terminal-pro-B-type-natriuretic-peptide, *ACEI/ARB* Angiotensin-converting enzyme inhibitors or Angiotensin receptor blockers, *BLR* Blood urea nitrogen to left ventricular ejection fraction ratio

**Fig. 6:**
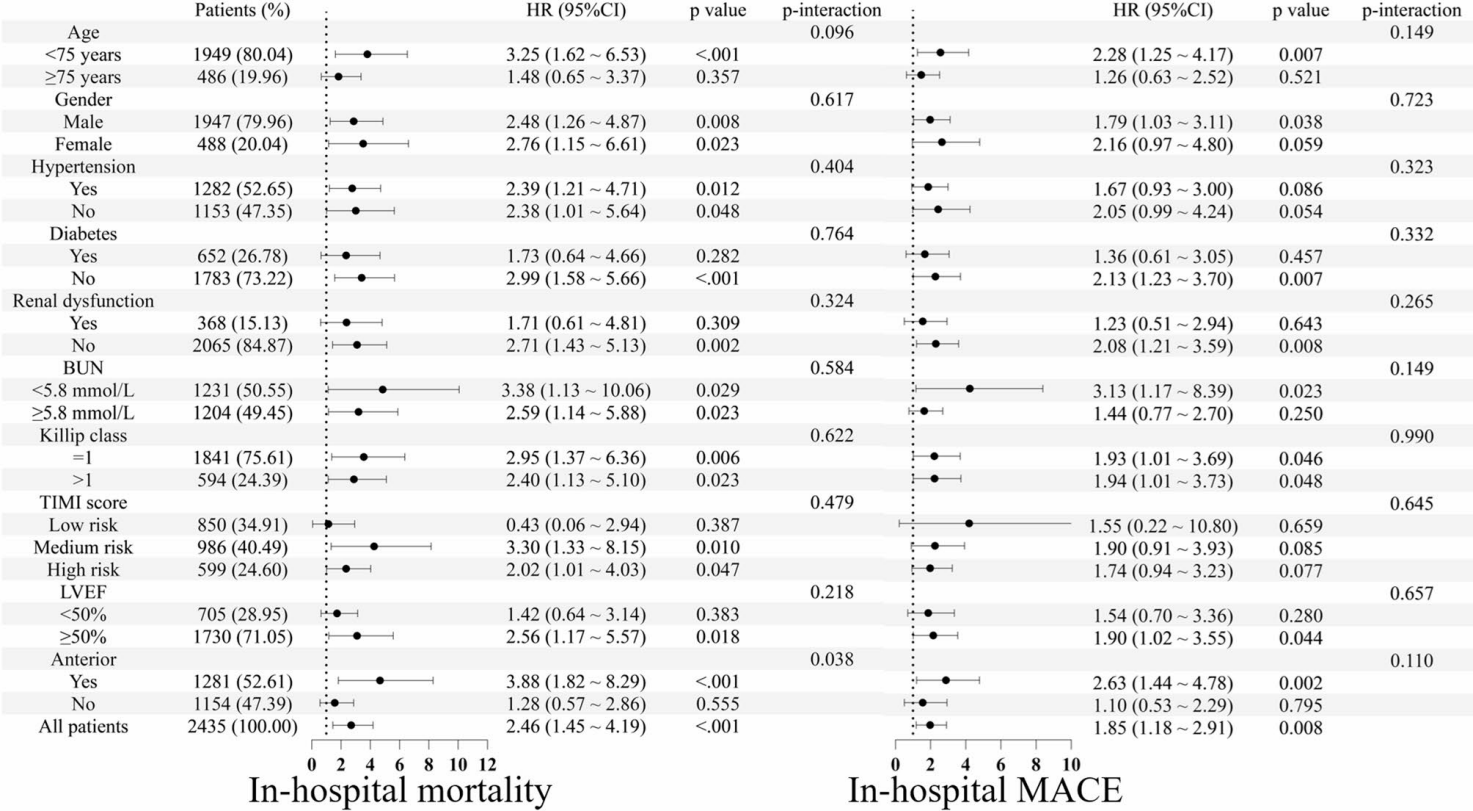
Subgroups analysis of in-hospital mortality and MACE in STEMI patients (MACE: major adverse cardiovascular events, STEMI: ST-elevation myocardial infarction, BUN: blood urea nitrogen, LVEF: left ventricular ejection fraction)

For a more detailed analysis of the association between BLR and in-hospital all-cause mortality and MACE in patients with different clinical profiling, subgroups were analyzed (Fig. 6). For in-hospital mortality, the effect of BLR on different subgroups were consistent except in anterior vs. non-anterior MI with more significant in patients with anterior MI compared with non-anterior MI (HR = 3.88, 95%CI 1.82, 8.29, p-interaction = 0.038). For in-hospital MACE, the effect of BLR on different subgroups were consistent (all p-interaction > 0.05).

## Discussion

The main findings of the present study are as follows: firstly, in STEMI patients, higher BLR level was associated with higher in-hospital mortality and MACE incidence, and a BLR level higher than 12.54 was an independent risk factor for in-hospital all-cause mortality and MACE; secondly, the predictive value of BLR was significantly higher than BUN, LVEF, creatinine, NT-proBNP, and TnI and was comparable to GRACE score and TIMI scores; thirdly, the effects of BLR on different clinical profiles were consistent but more obvious in patients with anterior MI. Our present study demonstrated the possibility of BLR as a biomarker for prognostic evaluation in STEMI patients.

Currently, TIMI score [7] and GRACE score [8] are the two scores widely used for risk stratification in patients with STEMI. However, the sophisticated Algorithm of these risk scores limited their bedside use, e.g. TIMI score includes seven variables as age, blood pressure, heart rate and Killip class et., while GRACE score includes even more variables that need to be calculated through the calculator. More importantly, the predictive efficacy of these two scores is modest. Previous studies demonstrated that the AUCs of TIMI score and GRACE score for predicting the short-term outcome in patients with STEMI were 0.779 and 0.77, respectively [7, 8]. In our study, the prognostic value of TIMI score and GRACE score was Also modest with the AUCs of 0.770 and 0.784, respectively. Therefore, a simple and effective tool for risk stratification in patients with STEMI is urgently required.

BLR was initially designed by Kiris et al. [13], who used this index to evaluate the risk of contrast induced nephropathyin (CIN) in patients with acute coronary syndrome undergoing PCI and found out BLR was significantly higher in those who developed CIN, and as a continuous variable, BLR was independently associated significantly increased risk of CIN (OR 10.59, 95% CI 2.803–40.070, *p* = 0.001). Subsequent studies demonstrated that in patients with coronary artery disease, BLR was shown to be associated with all-cause mortality and decompensated heart failure incident in patients with stable angina pectoris, and the predictive value of BLR was superior to BUN or LVEF alone [12]. This finding was further confirmed in patients receiving coronary artery bypass grafting, and a remarkable elevated BLR predicted increased risk of long-term mortality and new-onset decompensated heart failure [14]. Recently, Ozkan et al. [15] found that in patients with acute heart failure, BLR was shown to be superior to BUN or LVEF for predicting the outcome. Similarly, our recent study also revealed that in patients with AMI complicated by cardiogenic shock, higher BLR was associated with poor short-term prognosis and the predictive value of BLR was superior to BUN or LVEF alone [19]. The current study extended previous findings, indicating that BLR was a potential useful marker from high-risk patients with cardiogenic shock to general STEMI patients, demonstrating the prognostic value of BLR in a broader patient population.

In the setting of STEMI, a series of pathophysiological process, such as reduced cardiac output, systemic congestion, the activation of systemic vasoconstriction, diuretics use, and the administration of contrast media during revascularization could impair renal function [20], which in turn, aggravates cardiac dysfunction via activation of renin-angiotensin-aldosterone system (RAAS) and finally CRS develops with a vicious cycle between renal and cardiac dysfunction [9]. Therefore, an adequate assessment of the cardiorenal interaction in the context of STEMI received PCI is of great importance. Our present study showed patients with higher BLR had more high risk clinical characteristics, such as older age, more morbidities and complications on admission, which were variables include in higher TIMI and GRACE scores; however, multivariate Cox regression suggested high BLR remained an independent risk factor for in-hospital mortality and MACE, indicating that BLR has independent predictive value in STEMI patients.

Both BUN and creatinine reflect the renal function; however, BUN may increase before the elevation of creatinine or the decrease of glomerular filtration rate, because the overactivation of the sympathetic nervous system and RAAS could enhance the reabsorption of BUN in proximal tubular in the early stage of renal hypoperfusion [21, 22]; therefore, elevated BUN level not only reflects renal dysfunction but more importantly, indicates the neurohormonal activation, which has widely been demonstrated to be related with the prognosis in patients with AMI [23–25]. Actually, previous studies have confirmed the prognostic value of elevated BUN in patients with AMI [26–29]; furthermore, the predictive value of BUN was shown to be superior to that of creatinine [10]. Therefore, as shown in the present study, the AUC of BUN was significantly higher than that of creatinine. LVEF is the most important index to describe the left heart systolic function, AMI usually causes the decrease of LVEF [30], especially in the setting of anterior MI, which directly cause pump failure. Subgroup analysis in our present study also revealed the impact of BLR was more significant in patients with anterior MI compared with non-anterior MI, possibly due to the significant decrease in LVEF in patients with anterior MI. Similarly, LVEF is an independent factor associated with the prognosis in patients with STEMI [1, 31–36]. Left ventricular dysfunction may develop at the onset of STEMI due to extensive myocardial necrosis or later during the process of ventricular remodelling [37]. BLR takes the two important prognostic factors together and in our present study, BLR exhibited superior predictive value compared with BUN or LVEF alone.

Although the precise molecular mechanisms why BLR is associated with the short-term prognosis remains unknown, multiple neurohumoral and inflammatory pathways are involved in CRS. Firstly, left ventricular dysfunction causes prerenal hypoperfusion, which activates sympathetic nervous system, RAAS, and arginine vasopressin secretion, leading to fluid retention, increased preload, and worsening pump failure [38]. Secondly, when AMI occurs, a series of inflammatory biomarkers such as white blood cell, C-reactive protein, interleukin−6, reactive oxygen species are elevated [39], which have direct cardiodepressant effects causing a reduction in LVEF [9]. What’s more, activated systemic inflammation could induce renal endothelial dysfunction and have been confirmed to be involved in the development of acute kidney injury after AMI and are associated with poor clinical outcome [40, 41]. In addition, several other pathways that exacerbate cardiac or kidney injury, including activation of the sympathetic nervous system, imbalance in the proportion of reactive oxygen species/nitric oxide production, and persistent RAAS activation [42], further induces both cardiac and renal dysfunction and causes a vicious cycle, leading to astounding morbidity and mortality.

In contrast to other risk scores, BLR focused on the interaction between the two most important and commonly damaged organs (heart and kidney) after STEMI. Our present study shows the prognostic value of BLR was comparable to or even higher than that of TIMI score or GRACE score. Therefore, as a convenient, effective and low cost index, BLR provides a tool for risk stratification in patients with STEMI and should be taken into risk stratification model.

Some limitations in our study should be addressed. First, as an retrospective observational study, some unmeasured and unknown confounding factors cannot be well controlled. Second, we only analyzed the association between admission BLR with in-hospital outcome; however, the dynamic changes of BLR during hospitalization was unavailable. The dynamic change of BLR could provide more prognostic value. Third, patients with chronic kidney disease that could cause elevated BUN, which may compromise the interpretability of BUN-driven risk estimation although we conducted subgroup analyses based on BUN and the status of renal dysfunction and the effect of BLR was consistent between patients in the different subgroup patients. Nevertheless, residual confounding due to baseline renal impairment may affect the interpretation of the results. Fourth, we only evaluated the association between BLR with short-term outcome, the prognostic value of BLR for long-term outcome remained unclear. Finally, no mechanistic biomarkers (e.g., inflammatory or neurohormonal mediators) were incorporated into the analysis to substantiate the hypothesized pathways linking BLR to adverse outcomes due to the retrospective design and data on these aspects were not available. Future prospective studies incorporating relevant biomarkers are warranted to explore the underlying mechanisms and to further substantiate the prognostic value of BLR. In addition, this was a single-center retrospective study, and the interpretation and generalization of the results should be made with caution. Therefore, multicenter prospective studies are needed to validate the prognostic value of BLR, including its impact on long-term outcomes.

## Conclusion

Admission BLR provided important prognostic information for STEMI patients especially in patients with anterior MI and the predictive validity of BLR was comparable even better than traditional risk scores. An admission BLR higher than 12.54 was significantly associated with increased risk of in-hospital mortality and MACE. This easily accessible index might be promising for early risk stratification in STEMI.

## Data Availability

The data that supports the findings of this study are available from the corresponding author upon reasonable request.

## Abbreviations

STEMI: ST-elevation myocardial infarction
BUN: Blood urea nitrogen
LVEF: Left ventricular ejection fraction
BLR: Blood urea nitrogen to left ventricular ejection fraction ratio
MACE: Major adverse cardiovascular events
ROC: Receiver operating characteristic
AUC: Area under the curve
NT-proBNP: N-terminal pro-brain natriuretic peptide
ACEI: Angiotensin-converting enzyme inhibitors
ARB: Angiotensin receptor blockers
HR: Hazard ratio
CI: Confidence interval
PCI: Percutaneous coronary intervention
AMI: Acute myocardial infarction
CRS: Cardiorenal syndrome
TnI: Troponin I
SD: Standard deviation
CIN: Contrast induced nephropathyin
RAAS: Renin-angiotensin-aldosterone system
